# Assessment of dog owner adherence to veterinarians’ flea and tick prevention recommendations in the United States using a cross-sectional survey

**DOI:** 10.1186/s13071-017-2217-2

**Published:** 2017-06-06

**Authors:** Robert P. Lavan, Kaan Tunceli, Dongmu Zhang, Dorothy Normile, Rob Armstrong

**Affiliations:** 1 Outcomes Research, Animal Health, Center for Observational and Real-World Evidence, Merck & Co., Inc, Kenilworth, NJ USA; 2 MSD Animal Health, 2 Giralda Farms, Madison, NJ USA

**Keywords:** Compliance, Adherence, Flea, Tick, Dog, Fluralaner, Ectoparasites

## Abstract

**Background:**

Adherence to a prescribed therapeutic regimen is a critical factor for achieving medication effectiveness and therefore treatment success. In the case of companion animal ectoparasite control, suboptimal owner adherence to medication recommendations is thought to be a common cause of treatment failure, and previous reports have found pet owners applying an average of 4.0–4.6 monthly flea and tick treatments per year to their dogs. This study investigated: US veterinary hospital self-reported flea and tick prevention recommendations; dog owner recollection of these recommendations; dog owner opinion on flea/tick recommendations and estimated owner flea and tick medication adherence based on veterinary hospital purchase records.

**Results:**

Veterinarians at 24 veterinary hospitals in 4 United States regions provided their flea and tick prevention recommendations. Five hundred fifty-nine dog owners, clients of the 24 hospitals, completed a survey evaluating their recollection of the hospitals’ recommendations and their opinions regarding required treatment frequency. Almost all veterinary hospitals in this study recommended 12 months of flea and tick prevention but only 62% of participating dog owners recalled this recommendation. The average owner response was that their dogs require 10.5 months of flea and tick prevention annually. Owner opinions were significantly different among U.S. regions with pet owners in the northeast U.S. believing that they needed significantly less canine flea and tick protection than pet owners in other parts of the United States. The estimated actual flea and tick prevention coverage was 6.1 months based on owner medication purchases over a 12-month period.

**Conclusions:**

In the United States, dog owner opinions and actions show that their flea and tick treatment adherence falls short of veterinarians’ recommendations.

## Background

Parasite prevention medicines are more likely to be effective, and thereby protect the treated animal, when they are used according to appropriately prescribed re-treatment intervals. “Compliance” and “adherence” are commonly and interchangeably used to describe how well the actual use of a medication agrees with the prescription instructions. However, these are not identical terms. “Compliance” refers to how closely the patient follows the prescriber’s advice, and use of this term carries the negative idea that any failure in medication use is the patient’s fault. “Adherence” indicates that the patient is making an effort to maintain a prescribed regimen, and this more positive interpretation is increasingly used for reporting medication use patterns [1]. Full adherence to a prescribed regimen is achieved when the correct medication is administered at the correct dose, at the correct time, over the complete dosing interval. Non-adherence results in suboptimal pharmacotherapy, and is a potential contributor to negative outcomes like parasite resistance or disease progression, decreased treatment satisfaction, reduced patient quality of life, and increased treatment costs [2–4]. A meta-analysis of 569 studies reporting adherence to physician-prescribed medical treatment between 1948 and 1998 found that patient non-adherence ranged from 0 to 95% with an average non-adherence rate of 24.8% [5]. Adherence to a therapeutic regimen is multifaceted, with failure to adhere attributed to more than one factor [1, 6, 7]. A review of ectoparasite resistance to medications concluded that the most likely cause of suspected lack of insecticide/acaricide efficacy is treatment deficiency rather than resistance [8].

Techniques used to measure adherence to medication recommendations have included patient self-reported methods (verbal interviews, diary studies or questionnaires), indirect non-self-reported methods (administrative claims, electronic monitors, pill count or canister weight) or direct non-self-reported methods (plasma drug concentration, biological marker or directly observed therapy) [9, 10]. Patient surveys are an example of a self-reported adherence measure that has the advantage of obtaining information from the patient’s perspective, including non-adherence reasons. Patient surveys also carry the disadvantage of patient recall and bias. There is no gold standard for measuring medication adherence but patient surveys are commonly used [11].

Studies on human patient adherence to prescribed treatment regimens generally report an inverse relationship between medication adherence and dosing frequency, with significantly higher adherence rates reported for medications with a longer duration of action and therefore decreased dosing frequency [12–15]. This inverse link between adherence and dosing frequency has been demonstrated across a variety of drug classes [16, 17]. This relationship is partially responsible for the current trend toward longer-acting formulations in human medicine [18, 19]. There is evidence for a similar relationship between adherence and dosing frequency in veterinary medicine. Pet owners administering short-term antimicrobials to dogs were nine times more likely to be compliant with a once or twice a day dosing regimen compared to a three times daily dosing regimen [20]. Longer-acting veterinary formulations are also becoming more widely available including extended duration heartworm preventives and antimicrobials. Over the past 20 years, monthly (or 4 week) re-treatment has been the standard dosing interval for most flea and tick treatments for dogs. In 2014, a systemic treatment option was introduced that offered up to 12 weeks of flea and tick protection following administration of a single oral chewable dose of fluralaner (Bravecto®, Merck Animal Health, Madison, NJ USA). There are currently long acting flea/tick collars available for dogs in the United States, that have label indications for multi-month efficacy (flumethrin and imidicloprid, 8 months; deltamethrin 6 months). This study looks at adherence that is the result of a single pet owner decision regarding periodic product administration that cannot be undone by accidental factors (like the loss of a collar).

The optimal measure of adherence is to record the actual administration of a dose of the flea/tick medication to the dog; however, this degree of supervision was impractical. As a surrogate, this study surveyed owners currently treating their dogs with oral fluralaner, a prescription-only chew containing the long-acting systemic insecticide and acaricide, for flea and tick protection. This inclusion criterion was used to standardize owner responses to treatment and to reduce the potential that owners were purchasing over-the-counter (OTC) flea and tick treatments that could not be monitored. This approach allowed evaluation of sales recorded in the practice management database to provide a more accurate estimate of treatments purchased. Fluralaner purchases were used as a surrogate measure to estimate the number of doses administered to dogs in the practice.

In summary, the objectives of this study were to use survey techniques to: describe veterinary recommended flea and tick treatment protocols; assess owner recollection of veterinary recommendations for flea and tick prevention; and record owner opinions regarding the required period of protection. In addition, an estimate of actual owner adherence to administering flea and tick protection was assessed through a detailed review of flea and tick treatment purchases from selected veterinary hospital records.

## Methods

Twenty-four participating veterinary hospitals were selected representing four United States geographical regions: northeast, central, south and west (Table 1). Staff veterinarians at each participating veterinary hospital were interviewed to document the hospital’s flea and tick prevention recommendations. Veterinarians’ responses were cross-tabulated by gender, years in practice, number of months of flea protection recommended in a year, number of months of tick protection recommended in a year, and geographic region.

**Table 1:** Regional distribution of survey participants

US region	No. of hospitals (%)	US States	No. of enrolled dog owners
Northeast	4 (17)	NY, OH, PA	92
South	9 (38)	AL, FL, GA, TX	191
Central	8 (33)	AR, IL, KS, KY, MO, TN	192
West	3 (13)	CA, HI	84
Total	24		559

*Abbreviations*: *AL* Alabama, *AR* Arkansas, *CA* California, *FL* Florida, *GA* Georgia, *HI* Hawaii, *IL* Illinois, *KS* Kansas, *KY* Kentucky, *MO* Missouri, *NY* New York, *OH* Ohio, *PA* Pennsylvania, *TN* Tennessee, *TX* Texas

Each participating hospital identified at least 20 dog owners to complete a flea and tick prevention experience survey. For enrollment in the survey, owners were required to have visited the hospital for any reason (for example, an office visit or to pick up pet supplies) during the survey period between April 1, 2016 and June 15, 2016. Owners were interviewed either on the day of their hospital visit or by phone within 1 week after the hospital visit. The enrollment criteria were designed to identify households with an individual dog that was currently receiving fluralaner for flea and tick prevention based on the owner’s purchase history showing that the dog should be currently receiving this flea and tick treatment. Enrollment was restricted to either single-dog households or households where the identified dog received a different dosage size than all other household dogs, to maximize the chance that fluralaner purchases were administered to the individual dog. To avoid pet owners with limited fluralaner administration experience, dogs must have been prescribed at least two prior doses.

Dog owners meeting the enrollment criteria were given a study explanation sheet and asked to complete a flea and tick prevention survey administered by a practice employee. Survey results were cross-tabulated using the dog and pet owner demographic questions to compare the responses and assess the significance of observed interrelationships.

Clinics that administered the pet owner survey were asked to provide summaries of all fluralaner transaction data from all clients (not just study participants) for the 1 year period April 2015–March 2016. This 1 year period represented the full calendar year immediately prior to the initiation of the study. Six clinics located in California, Florida, Kansas, Kentucky and New York provided these records. Transaction records were used to calculate the average number of fluralaner doses purchased for each dog in the designated 12 month period. This information was converted to weeks and months of fluralaner coverage.

Descriptive statistics such as frequencies and percentages for categorical variables and mean (standard deviation, SD) for continuous variables were used to describe the characteristics of the pet owners and dogs. Tests of analysis of variance (ANOVA) and Tukey’s *post-hoc* multiple comparison test were used to test the differences in pet owners’ opinion of months of flea and tick treatment needed for their dogs across regions. Values of *P* < 0.05 were considered statistically significant. All statistical analyses were performed using SAS version 9.3 (SAS Institute, Cary, NC).

## Results

Twenty-four veterinary hospitals spread across 4 different US regions participated in the study (Table 1). The majority of participating veterinary hospitals recommended 12 months of flea (23/24) and tick (23/24) protection per year, and the one dissenting hospital is not the same for the flea (a hospital in the northeast region) and tick (a hospital in the central region) recommendation. Other than the dissention noted, veterinary flea and tick control recommendations were the same across the 4 regions of the United States and were not affected by the number of years the participating veterinarians worked in practice (flea control *F*_(3,22)_ = 1.48, *P* = 0.247; tick control *F*_(3,22)_ = 0.50, *P* = 0.685). A summary of the pet owner demographics is included in Table 2 and the dog demographics is in Table 3.

**Table 2:** Characteristics of participating dog owners

Pet owner characteristic	Overall sample (*N* = 559)
Age range in years, *n* (%)	
Not known	63 (11.3)
10–49	232 (41.5)
50–69	223 (39.9)
70+	41 (7.3)
Gender, *n* (%)	
NA	29 (5.2)
Female	390 (69.8)
Male	140 (25.0)
Years as primary caregiver for the dog,	
*n* (%)^a^	542 (97.0)
Mean (SD)	5.5 (3.6)
Median (Range)	5.0 (0.4–16.0)
Participating dog owners by US Region, *n* (%)	
Central	192 (34.3)
Northeast	92 (16.5)
South/Southeast	191 (34.2)
West	84 (15.0)
Direct experience with fleas, *n* (%)	
No	314 (56.2)
Yes	245 (43.8)
Direct experience with ticks, *n* (%)	
No	357 (63.9)
Yes	202 (36.1)
Pet owners that had used other products by region, *n* (%)	
Overall	406 (72.6)
Central	134 (69.8)
Northeast	67 (72.8)
South/Southeast	141 (73.8)
West	64 (76.2%)

*Abbreviation*: *NA* Data not available

^a^ *n* (%) number of non-missing values and %

**Table 3:** Characteristics of dogs enrolled in the study (*n* = 559 except where noted)

Dog characteristic		
Gender, *n* (%)	Male	288 (51.5%)
	Female	270 (48.3%)
Neutering status, *n* (%)	Neutered	493 (88.2%)
	Not neutered	62 (11.1%)
Age (years; *n* = 557)	Mean ± SD	6.1 ± 3.6
	Median (Range)	6.0 (0.6–16.0)
Weight (lbs; *n* = 554)	Mean ± SD	41.4 ± 30.4
	Median (Range)	35.0 (3.8–200.0)
Dogs that participate in outdoor activities, *n* (%)	Yes	462 (86.2%)
	No	97 (17.4%)
Daily outdoor time by region, hrs (Mean ± SD)	Northeast (*n* = 90)	4.1 ± 4.3
	Central (*n* = 190)	3.3 ± 3.7
	South (*n* = 185)	5.3 ± 6.3
	West (*n* = 83)	3.8 ± 4.0
	Average (*n* = 548)	4.2 ± 4.9

*Abbreviation*: *SD* standard deviation

The 559 enrolled dog owners were 70% female, 25% male, and 5% unspecified (a few owners either did not answer the question or circled multiple responses). Pet owner age was requested in 10 year age blocks, and ranged from 10 to 19 years to 80–89 years with the 50–59 year age block selected most frequently. Forty one percent of owners had seen fleas on their dog and 31% had seen ticks on their dog prior to starting treatment. Just over half of owners (342/559, 62%) accurately recalled their veterinarian’s 12 months of flea and tick protection recommendation, while 14% (76/559) underestimated the recommended number of months and 25% (138/559) had no recollection of their veterinarian’s recommendation. Dog owners were asked for their opinion regarding the number of months of flea and tick coverage their dog should receive, and the average response was that dogs require 10.6 months of coverage during the year. Most owners (350/478, 73%) believed their dog needed 12 months of flea and tick protection over the year, while a few (49/478, 17%) believed their dog needed 6 months or less of coverage (Table 4). The Northeast region had the smallest proportion of dog owners (46/85, 54%) who believed their dog required 12-months of flea and tick protection while the other regions averaged 77% (304/393). The number of months of flea/tick protection preferred by dog owners was significantly different across regions (*F*_(3474)_ = 5.06, *P* = 0.002). The *post-hoc* Tukey’s test suggests that the number of months of flea/tick protection preferred by dog owners was significantly less in the Northeast region compared to all other regions (Table 5).

**Table 4:** Dog owner opinions on the need for canine flea/tick protection in the US

US region	No. (%) of responses on treatment duration needed by US region				
	0–5 months	6 months	7–11 months	12 months	Average number
Northeast (*n* = 85)	6 (7)	20 (24)	13 (15)	46 (54)	9.5
South (*n* = 156)	14 (9)	9 (6)	8 (5)	125 (80)	10.7
Central (*n* = 162)	7 (4)	14 (9)	21 (13)	120 (74)	10.7
West (*n* = 75)	3 (4)	6 (8)	7 (9)	59 (79)	10.9
Overall (*n* = 478)	30 (6)	49 (10)	49 (10)	350 (73)	10.6

**Table 5:** Regional paired comparisons of pet owners’ opinion of flea and tick treatment needed for their dogs^a^

Comparison	Difference between means	*P*-value
West *vs *South	0.13	0.982
West *vs* Central	0.15	0.978
West *vs* Northeast	1.35	0.008
South *vs* Central	0.01	1
South *vs* Northeast	1.21	0.004
Central *vs* Northeast	1.20	0.005

^a^Tukey’s test results following the ANOVA

Transaction records collected from 6 veterinary hospitals included 9370 purchases of fluralaner for 5290 dogs between April, 2015 and March, 2016. Owners purchased an average of 2.18 doses of fluralaner for their dog, which translates to 26.2 weeks or 6.1 months of fluralaner coverage annually (Table 6). Only 13% of dog owners in this analysis purchased 4 doses of Bravecto to provide approximately 1 year of coverage.

**Table 6:** Summary of fluralaner purchases at six US veterinary hospitals

State	Dogs (*n*)	Mean doses per dog	Average annual flea and tick coverage	
			Weeks	Months
CA	994	1.84	22.1	5.1
KY	964	1.98	23.8	5.5
KS	1306	2.54	30.5	7.1
FL	818	2.22	26.6	6.2
NY	557	1.81	21.7	5.1
NY	654	2.56	30.7	7.1
Total/Mean	5293	2.18	26.2	6.1

*Abbreviations*: *CA*California, *FL* Florida, *KS* Kansas, *KY* Kentucky, *NY* New York

## Discussion

U.S. veterinarians recommended that pet owners protect their dogs for a full year against fleas and ticks which is consistent with recommendations of the Companion Animal Parasite Council (CAPC) [21]. In our study, over half the dog owners (62%) recalled their veterinarian’s 12 month recommendation and, perhaps surprisingly, a larger proportion of owners independently held the opinion that their dog required 12 months of flea and tick protection (73%). Dog owner fluralaner purchases, likely the most accurate estimate for adherence, suggest that the average dog owner actually purchased slightly over 6 months of fluralaner coverage, while 13% purchased enough medication (four doses) to fully adhere to the most common veterinary recommendation. These results show a sequential decrease in flea and tick control duration from veterinarians’ recommendations, to dog owners’ beliefs and then to dog owners’ actions.

The survey did not determine the reasons for reduced dog owner adherence. Possible reasons could be: a belief that protection is not needed at some times of the year; forgetfulness or competing financial priorities. Northeast dog owners were more likely than other region owners to report their dogs needed less than 12 months of flea/tick protection although veterinarian protection duration recommendations were consistent across regions. Therefore, northeast dog owners may benefit from additional education and support regarding the importance of year round flea and tick protection.

Medication adherence depends on successfully completing a sequence of steps: clear communication of the recommendation; agreement between owner and veterinarian on the importance of treatment; communication of a consistent recommendation by all hospital personnel; owner purchase of sufficient doses of an effective treatment; and correct timing and administration of prescribed doses to deliver the recommended protection interval. The literature in human medicine has generally reported improvement in medication adherence with prescriptions for longer-acting medications and therefore medications with a decreased dosing frequency [12–15]. For many years, the standard for US flea and tick parasiticides was to require retreatment approximately every 4 weeks. Reports of owner adherence during the past few years estimated that dog owners administered doses to deliver between 4.0 and 4.6 months of flea and tick protection per year, [22, 23] or approximately one third of the year. Fluralaner, approved in 2014, was the first systemic flea/tick parasiticide with a 12 week retreatment interval. Examination of prescription records found that dogs prescribed fluralaner received an estimated 6.1 months or protection per year, or about half the year. This suggests that longer retreatment intervals might contribute to improvement in dog owner adherence. Future studies measuring adherence rates of pet owners prescribed either short or long retreatment interval flea and tick products is needed to confirm this observation.

Accurate recall of a veterinary recommendation requires initially that the veterinarian clearly provides a comprehensible and distinct recommendation that is understood by the owner. Odds of client adherence were 7 times greater when the owner received a clear recommendation compared with owners who received an ambiguous recommendation in an investigation of videotaped veterinarian-client-patient interactions [24]. Next, the owner needs to remember the recommendation among all the other information communicated during the veterinary visit. A written summary of office visit recommendations, followed at the appropriate time by a reminder from the clinic will help the owner remember to time retreatment correctly.

Dog owners need to understand the significance of, and agree with, veterinarian recommendations before acting on them. In this study, most owners agree that their dog requires flea and tick protection but on average owners thought their dogs needed 1.4 fewer months of protection per year than recommended by veterinarians. Therefore, veterinarians may want to refine their practice communication strategies to improve owner adherence. A comprehensive hospital communication plan will effectively support the recommended treatment strategy, and steps could include: ensuring all hospital staff know the recommendation; providing clear written instructions to send home with the owner; post-visit follow-up communication from the practice; use of social and digital media to communicate the hospital position; and use of reminders to support hospital visits and re-treatments [25].

In our study, medication adherence has been estimated through analysis of purchase data over 12 months. The calculation may underestimate adherence if doses purchased prior to the study period are used in the 12 month study window. The calculation may also overestimate adherence if doses purchased were not administered or were given to a different dog.

A prior history of observing fleas and ticks could be expected to be a strong motivator for pet owners to adhere to prevention recommendations. However, effective flea and tick control may actually have a counter-productive effect because non-adherence of human patients is more common when the patient has no clinical signs for the disease being treated [12]. Owners may not be aware of the negative health effects that repeated flea and tick bites can cause in a dog and judge ectoparasitism as less serious than other health problems. This study found that the dog owner’s prior experience with fleas and ticks was not associated with their opinion on the duration of flea and tick protection required (*r*_(478)_ = 0.0433, *P* = 0.345).

## Conclusions

The required period of flea and tick protection decreases from veterinarians’ recommendation to owner belief to owner action. Owner adherence might improve with the use of a product with a longer retreatment interval. Additional research is needed looking at the transaction records of a large number of pet owners across multiple flea and tick products with different dosing schedules.

## Abbreviations

ANOVA: Analysis of variance
CAPC: Companion Animal Parasite Council
SD: Standard deviation
